# Effect of bread microstructure on internal liquid penetration using the lattice Boltzmann method combined with X-ray micro-computed tomography

**DOI:** 10.1016/j.crfs.2026.101380

**Published:** 2026-03-13

**Authors:** Hongling Zhou, Farshad Gharibi, Xueming Xu, Abdolreza Kharaghani, Dominique Thévenin

**Affiliations:** a School of Food Science and Technology, Jiangnan University, 1800 Lihu Road, Wuxi, 214122, Jiangsu, PR China; b Laboratory of Fluid Dynamics and Technical Flows, University of Magdeburg “Otto von Guericke”, Magdeburg, D-39106, Germany; c Thermal Process Engineering, University of Magdeburg “Otto von Guericke”, Magdeburg, D-39106, Germany

**Keywords:** Lattice Boltzmann simulation, Bread microstructure, Permeability, Structural parameters, Porous media

## Abstract

Understanding the influence of food matrices on the penetration process is essential for optimizing filling and spreading techniques in food processing. In this study, an enhanced lattice Boltzmann method coupled with X-ray micro-computed tomography scanning is used to simulate fluid dynamics within the real microstructure of bread at the pore scale. The influence of micro-structural characteristics of bread (porosity, connectivity, pore size distribution, tortuosity) on liquid penetration is investigated. The numerical model is validated using three benchmarks to ensure its applicability and assess accuracy for heterogeneous porous media. The results indicate that relying solely on porosity or effective porosity to predict permeability is not sufficient, as no simple correlation is observed between permeability and porosity. Investigating the internal flow dynamics suggests that higher connectivity, particularly along the main flow direction, is identified as the dominant factor controlling permeability by offering further paths for fluid movement. In addition, details of the pore structure also play an important role, especially when many small broken holes are observed. Those constitute bottlenecks that restrict fluid flow and significantly influence the global flow features. The median pore size ($D_{50}$) is identified as a representative measure of flow capacity. In contrast, tortuosity and the number of junctions show only negligible correlations with permeability.

## Highlights

• X-ray micro-tomography was utilized to analyze the microstructure of bread.
• An enhanced lattice Boltzmann method was employed to capture pore-scale liquid penetration in bread.
• Key pore structural properties were quantified and related to permeability prediction.
• (Effective) porosity alone is inadequate for accurate permeability prediction.
• Topological characteristics strongly influence fluid flow in bread.

## Graphical abstract

**Figure:**
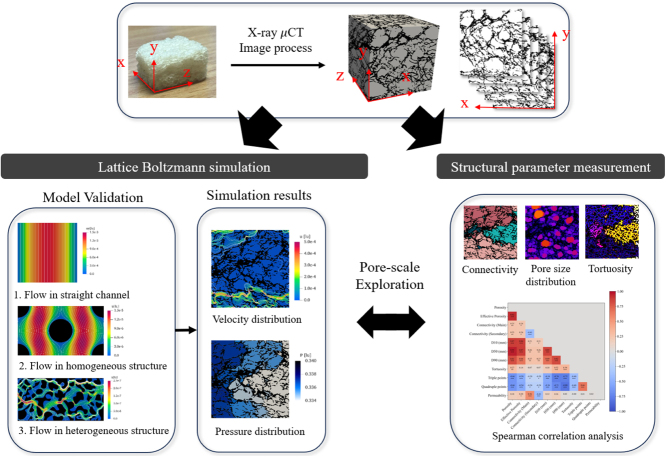

## 1 Introduction

Filling and spreading techniques, commonly applied to sauces and similar food products, are widely used and play a crucial role in food processing and consumption. Bread, as one of the most common carriers for sauces, spreads, and liquid fillings, is used in products such as sandwiches, filled pastries, and toast-based snacks. The penetration behavior of sauces in bread is not only a key determinant of texture, mouthfeel, and flavor release but also significantly impacts product stability and consumer acceptance. Depending on the specific application, different penetration levels are desired. The structural properties of bread, directly affect the penetration process by influencing fluid transport pathways, capillary forces, and resistance to flow. Variations in the microstructure lead to different penetration behaviors, which ultimately determine the distribution and retention of sauces within the bread matrix. Therefore, understanding the relationship between bread microstructure and penetration dynamics is essential for optimizing product formulations and processing techniques.

In order to address this challenge, the conventional experimental approach for studying fluid penetration in porous food matrices typically involves numerous tests. However, knowledge of all relevant experimental properties is still limited, preventing further progress in understanding how food structures can impact processing conditions and interactions with a liquid (Warning et al., 2014). Furthermore, acquiring experimentally detailed fluid flow data in bread remains very challenging and costly (Li et al., 2023), in particular when considering the swelling process and resulting deformations. To overcome these limitations of experimental studies, a physics-based computational method is required. On the one hand, it provides a suitable source of complementary information to obtain details of the fluid flow at the pore scale, enriching experimental studies. On the other hand, such methods provide useful information about the flow path and other related properties that could be used to optimize food formulation and processing techniques.

During the last decades, the lattice Boltzmann Method (LBM) has emerged as a robust method of Computational Fluid Dynamics (CFD). LBM comes with significant advantages over traditional CFD techniques, such as a simple algorithm, easy implementation of complex geometries (e.g., using CT images), local computations, and inherent parallel efficiency. This also allows an easy implementation on graphical processing units (GPU), making LBM a popular technique for predicting flow behavior in porous media (Pan et al., 2006, Gharibi et al., 2018, Chen et al., 2022, Gharibi et al., 2024a, D’Orazio et al., 2023, Hou et al., 2025, Kuljabekov et al., 2023, Mohammadifar et al., 2021, Stockinger et al., 2024, Lautenschlaeger et al., 2022). In food manufacturing, the LBM has been used in various applications, such as analyzing the mechanical stresses experienced by food particles during transport (Marquardt et al., 2025), capturing the non-Newtonian flow behavior of chocolate emulsion during extrusion (Wu et al., 2025), simulating the freezing process of French fries (van der Sman, 2023), modeling heat and moisture transfer during bread baking (Hussein and Becker, 2010, Mack et al., 2011), drying (Panda et al., 2022) and extraction (Durán et al., 2015, Rosa et al., 2016, Rabi et al., 2020, Bertoldi et al., 2021). Specifically, for penetration processes in food matrices, LBM has been used for calculating the intrinsic permeability in apples and in rice heaps (Warning et al., 2014). LBM has also been employed to simulate the bread-baking process using simplified geometries (Hussein and Becker, 2010), addressing heat and mass transfer in dough, effective moisture transport, thermal conductivity, and diffusivity. So far, only a few studies have been conducted in this field, and all of them have used the standard Bhatnagar–Gross–Krook (BGK) LBM, which suffers from known limitations and inaccuracies in simulating processes in porous media (Gharibi and Ashrafizaadeh, 2020, Pan et al., 2006).

Bread is generally considered to be a solid foam structure, composed of a solid phase (cell walls) and a fluid phase (primarily air) (Rathnayake et al., 2018). To get accurate simulation results for the study of key properties of fluid flow in bread, one effective approach would be to combine micro-computed tomography (micro-CT or μCT) imaging, which allows for an in-depth characterization of bread microstructure at high resolution, with the lattice Boltzmann method, as done in the present study. Owing to the strong contrast between solid and fluid phase within bread, μCT can provide details of its cellular structure (Van Dyck et al., 2014, Cafarelli et al., 2014, Chen et al., 2021a). In order to describe and analyze this maze-like porous structure at the micrometer scale, various structural parameters, such as porosity, pore size distribution, connectivity, and tortuosity, have been proposed (Dadmohammadi and Datta, 2022, Gao et al., 2018, Demirkesen et al., 2014, Espinoza et al., 2015, Sheikh and Pak, 2015, Ghaitaranpour et al., 2025). These parameters have been employed to explain and evaluate the impact of bread production factors such as flour quality (Germishuys and Manley, 2021, Han et al., 2025), proofing conditions (Primo-Martín et al., 2010, Rossella et al., 2025), baking (Besbes et al., 2013), and storage (Chen et al., 2021b, de Lima et al., 2023). Indeed, Laurent et al. (2008) has already employed the extended Carman–Kozeny’s mode — which applies to porous media such as soils or expanded solid foams and incorporates indicators such as porosity and tortuosity. In addition, several fractal models have been proposed in recent years to improve this equation (Li et al., 2016, Xiao et al., 2021). However, the applicability of these approaches to bread remains uncertain due to its complex and heterogeneous structure, and a systematic and in-depth understanding of the mechanisms governing the interaction between bread structure and fluid flow is still lacking.

In this research, we integrate an enhanced LBM and micro-CT technology to reveal flow details in the real geometry of bread microstructure, and build a bridge between structural parameters and the penetration behavior of the liquid. Specifically, the behavior of the sauce is simplified as a Newtonian fluid with constant viscosity in this first study. Possible non-Newtonian properties and surface tension effects in a two-phase flow will be considered in later investigations. Both two-dimensional (2D) and three-dimensional (3D) simulations are performed in this study. The 2D model is employed to effectively capture flow behavior and structural features through systematic simulations, whereas 3D simulations provide direct insights into the pore-scale flow within real microstructures — at a much higher computational cost.

**Fig. 1:**
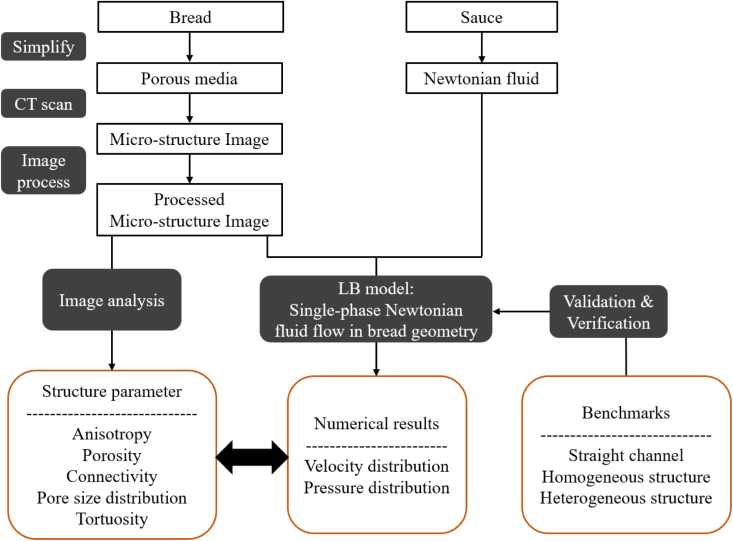
Structure of this study.

This work is implemented by developing an in-house GPU-based LBM solver, called Dena (Gharibi et al., 2024a, Gharibi and Ashrafizaadeh, 2020), to leverage parallelism on GPU. Specifically, permeability is employed as a key metric to quantify the penetration level, providing a direct measure of how easily fluid infiltrates the porous bread structure. Firstly, the correctness of our solver is verified and validated using three benchmarks. After ensuring that this method can handle simulations in the heterogeneous structure, it is used for simulating the penetration process in the real micro-CT bread image. Finally, the numerical results (velocity distribution, pressure distribution, permeability) are employed to explore the effect of structural parameters on the internal flow behavior. Such results are crucial for the development of new bread formulations and for optimizing standard manufacturing processes, ultimately leading to high-quality products. The structure of the study is shown in Fig. 1.

## 2 Methods

### 2.1 X-ray micro-tomography

In this study, we focused on Sandwich bread, composed of typical bread-making ingredients — wheat flour, water, yeast, and butter — due to its widespread consumption and structural uniformity, which make it a suitable sample for general studies on bread structure. The structure of the bread was imaged by micro-CT using a CT ProCon alpha 2000 (ProCon X-ray GmbH, Sarstedt, Germany). For these scans, a small sample of approximately 2 × 2 × 2 cm${}^{3}$ was prepared by cutting from the center of the bread loaf and mounted on the sample holder. The employed scanning parameters are summarized in Table 1.

**Table 1:** Summary of X-ray μCT scanning parameters.

Scanning parameters	Range
Voltage (kV)	50
Current (μA)	50
Rotation sector (°)	0–360
Exposure time (ms)	1000
Projections	1200
Resolution (voxel size) (μm)	18.7
Measurement time (h)	1.4
Bread sample size in the X-axis (cm)	2.19
Bread sample size in the Y-axis (cm)	2.34
Bread sample size in the X-axis (cm)	2.59
Number of pixels in the X-axis	1173
Number of pixels in the Y-axis	1253
Number of pixels in the Z-axis	1383

### 2.2 Image processing

**Fig. 2:**
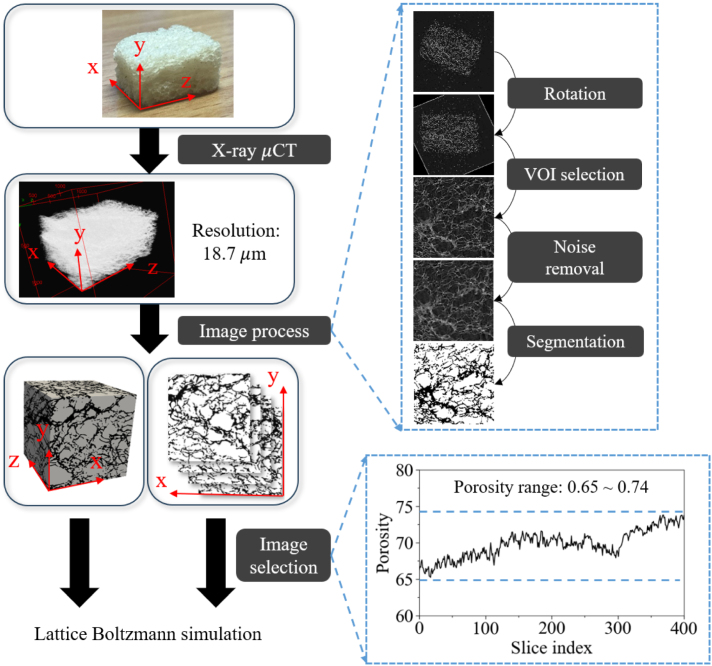
Workflow used for image processing and porosity measurement for the further investigation.

Following scanning of the samples, the obtained digital images were processed and analyzed using the open-source Fiji software (Schindelin et al., 2012). Before starting the image processing workflow, the 2D image stack was imported into Fiji, and the processing procedure was performed slice by slice on each 2D image. The image processing workflow consisted of several steps, including rotation, selection of the Volume of Interest (VOI), noise reduction, and finally segmentation, as illustrated in Fig. 2. Specifically, during VOI selection and in order to eliminate any edge effects and minimize structural damage caused by sample cutting (Besbes et al., 2013), and by considering the results of the representative elementary volume (REV) size test presented in the “Results and Discussion” section, a sub-volume of 400 × 400 × 400 voxels was selected from the center of the reconstructed 3D image — far from all boundaries. This corresponded to a final bread sample size of $7.5\times 7.5\times 7.5\;{\mathrm{mm}}^{3}$. Subsequently, a median filter, widely used in cereal-based structural studies (Besbes et al., 2013, Primo-Martín et al., 2010), was applied to enhance image quality by reducing noise. This was followed by the segmentation using Otsu’s method (Otsu et al., 1975) to separate the solid (bread structure) and the void (fluid area) regions into binary black-and-white parts. These processed images were subsequently employed to describe the real internal structure properties of bread and served as input data for the lattice Boltzmann simulations. Specifically, the processed 2D slices were directly used in the 2D simulations, while their stack was used as the input for the 3D simulations, with visualization performed in ParaView.

### 2.3 Image analysis and case selection

To quantitatively characterize the real microstructure of the bread, several parameters were determined from these processed 2D slices: porosity, connectivity, pore size distribution (PSD), and tortuosity.

Porosity was calculated as the ratio of gas volume to the total volume of the sample, represented in 2D images as the percentage of the white area relative to the total area, and in 3D images as the percentage of the white voxels relative to the total number of voxels. Connectivity was evaluated in Fiji by extracting individual pore regions using the “Analyze Particles” tool. Pore size distribution (PSD) analysis was performed using the “Local Thickness” tool in BoneJ plugin in Fiji (Dougherty and Kunzelmann, 2007, Hildebrand and Rüegsegger, 1997), which measures the diameter of the largest sphere that fits entirely within the pore space. Tortuosity is defined as the ratio between the actual path length through the structure and the straight-line (Euclidean) distance between the endpoints of that path:

(1) $$Tortuosity=\frac{L_{\text{Actual}}}{L_{\text{Euclidean}}}$$

where $L_{\text{actual}}$ is the total length of the skeletonized branch, and $L_{\text{Euclidean}}$ is the shortest straight-line distance between the two endpoints of that branch. In this study, the tortuosity was computed for each individual branch extracted from the skeletonized image using the BoneJ plugin in Fiji. The average tortuosity across all valid branches was then used as a representative measure of the structural complexity of the sample. In addition, two structural parameters, triple points (junctions with 3 branches) and quadruple points (4 branches), were also extracted from the same output.

After analysis, the porosity obtained from the 3D image was 0.698, and the porosity from the 2D slices was found to be between 0.65 and 0.74. Five groups were selected based on the porosity range and defined: P65, P67, P69, P71, and P73, where “P” means (cut-) Plane and the number represents the representative porosity of the group (P69: approximately 69% porosity).

### 2.4 Lattice Boltzmann method (LBM)

#### 2.4.1 Governing equation

To simulate the fluid flow through the porous domain, the lattice Boltzmann (LB) equation was used, which is written in discrete form as (Krüger et al., 2016):

(2) $$f_{i}\left(x+c_{i}\Delta t,t+\Delta t\right)=f_{i}\left(x,t\right)+\Omega_{i}+F_{i},$$

where i refers to the lattice directions, the number of those being determined by the selected lattice stencil, $f_{i}$ is the particle distribution function, and $c_{i}$ represents the discrete particle velocity vectors. The quantities x and t correspond to spatial location and time, with Δx and Δt denoting lattice spacing and time step, respectively, which are both set to unit values in lattice unit. On the right side of Eq. (2), $\Omega_{i}$ and $F_{i}$ respectively denote the collision operator and external force term. In the actual simulations, the LB equation was solved in two steps: collision and streaming, which are shown separately into Eqs. (3), (4).

Collision step:

(3) $$f_{i}^{*}\left(x,t+\Delta t\right)=f_{i}\left(x,t\right)+\Omega_{i}+F_{i}.$$

**Fig. 3:**
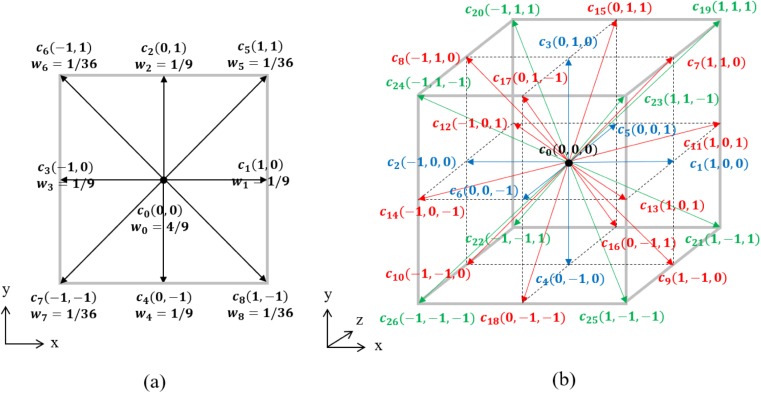
Velocity set and weight factors: (a) D2Q9 model and (b) D3Q27 model.

Streaming step:

(4) $$f_{i}\left(x+c_{i}\Delta t,t+\Delta t\right)=f_{i}^{*}\left(x,t+\Delta t\right).$$

where $f_{i}^{*}$ is the post-collision function. In this study, the D2Q9 and D3Q27 lattice stencils were employed for two-dimensional and three-dimensional simulations, respectively. The corresponding discrete velocity sets ($c_{i}$) and weight factors ($w_{i}$) are represented in Fig. 3. where $u=\left(u_{x},u_{y},u_{z}\right)$ is the macroscopic velocity vector.

Macroscopic parameters such as density ρ, fluid velocity $u=\left(u_{x},u_{y},u_{z}\right)$, fluid kinematic viscosity ν, dynamic viscosity μ, pressure P were calculated as follows:

(5) $$\rho \left(x,t\right)=\underset{i}{\sum }f_{i}\left(x,t\right),$$

(6) $$u\left(x,t\right)=\frac{\underset{i}{\sum }c_{i}f_{i}\left(x,t\right)}{\rho },$$

(7) $$\nu =c_{s}^{2}\left(\tau -\frac{\Delta t}{2}\right),$$

(8) μ=ρν,

(9) $$P=c_{s}^{2}\rho ,$$

where τ is the relaxation time and $c_{s}$ is the lattice speed of sound expressed as $c_{s}=\frac{1}{\sqrt{3}}\frac{\Delta x}{\Delta t}$.

In this study, a non-orthogonal central-moment collision operator was employed. First, the central moments k are defined in Eq. (10).

(10) $$k_{x^{m}y^{n}z^{p}}=\overset{Q-1}{\underset{i=0}{\sum }}f_{i}\;{\overset{¯}{e}}_{x,i}^{m}\;{\overset{¯}{e}}_{y,i}^{n}\;{\overset{¯}{e}}_{z,i}^{p},$$

where m=0,1,2,3, n=0,1,2,3, p=0,1,2,3, and $\overset{¯}{e}=\left({\overset{¯}{e}}_{x},{\overset{¯}{e}}_{y},{\overset{¯}{e}}_{z}\right)$ is defined as follows:

(11) $${\overset{¯}{e}}_{x,i}=e_{x,i}-u_{x},$$

(12) $${\overset{¯}{e}}_{y,i}=e_{y,i}-u_{y},$$

(13) $${\overset{¯}{e}}_{z,i}=e_{z,i}-u_{z}.$$

The detailed procedure for transforming the particle distribution functions $f_{i}$ into the central-moment space as $k_{i}$, follows the method described in Appendix A from the study of Gharibi and Ashrafizaadeh (2020).

Meanwhile, the equilibrium central moments $k_{i}^{eq}$ are obtained based on the same way, by transforming the equilibrium distribution functions $f_{i}^{eq}$ (Eq. (14)) into the central-moment space.

(14) $$f_{i}^{eq}\left(x,t\right)=w_{i}\rho \left[1+\frac{c_{i}\cdot u}{c_{s}^{2}}+\frac{{\left(c_{i}\cdot u\right)}^{2}}{2c_{s}^{4}}-\frac{u^{2}}{2c_{s}^{2}}+\frac{{\left(c_{i}\cdot u\right)}^{3}}{6c_{s}^{6}}-\frac{c_{i}\cdot u}{2c_{s}^{4}}u^{2}\right]$$

Then the post-collision central moments $k_{i}^{⋆}$ can be obtained as follows:

(15) $$k_{i}^{⋆}=k_{i}+\omega_{i}\left(k_{i}^{eq}-k_{i}\right)$$

Where $\omega_{i}$ denotes the relaxation frequency associated with the moment $k_{i}$. To ensure a fixed solid boundary location, $\omega_{i}$ are set up as Eq. (14) for the D2Q9 model and Eq. (14) for the D3Q27 model, according to the study of Ginzburg, 2006, Ginzburg, 2008.

D2Q9: $\omega_{0,1,2}=1$, $\omega_{3}=1.6$, $\omega_{4,5}=\frac{1}{\tau }$, $\omega_{6,7}=\frac{8\left(2-\frac{1}{\tau }\right)}{8-\frac{1}{\tau }}$, $\omega_{8}=1.8$.

D3Q27: $\omega_{4,\ldots ,8}=\frac{1}{\tau }$, $\omega_{10,\ldots ,16}=\frac{8\left(2-\frac{1}{\tau }\right)}{8-\frac{1}{\tau }}$, $\omega_{0,1,2,3,9,17,\ldots ,26}=1$.

Finally, the post-collision distribution functions $f_{i}^{⋆}$ can be obtained by multiplying the inverse transformation matrix with the post-collision central moments, according to the Appendix B of Gharibi and Ashrafizaadeh (2020). For details on the implementation of the model, the reader is referred to Gharibi and Ashrafizaadeh (2020).

Additionally, boundary conditions (BCs) play a crucial role to obtain the solution as they define the fluid behavior at the boundaries of the simulation domain. Two common boundary conditions were applied in our simulation: half-way bounce-back BC, and periodic BC. The former one describes the reflection of fluid particles when they meet a solid surface, which has the benefits of easily handling complex and irregular walls in porous materials, see Chen et al. (2020). While the latter one is used to simulate the situation of a periodic flow, for instance when the fluid leaving the outlet re-enters from the inlet. It is worth noting that the pore structures on opposite boundaries are generally not identical. To address this, one ghost layer was introduced at each periodic boundary, and the periodic BC was implemented by copying the particle distribution functions from one boundary to the corresponding ghost layer on the opposite boundary.

#### 2.4.2 Implementation

**Fig. 4:**
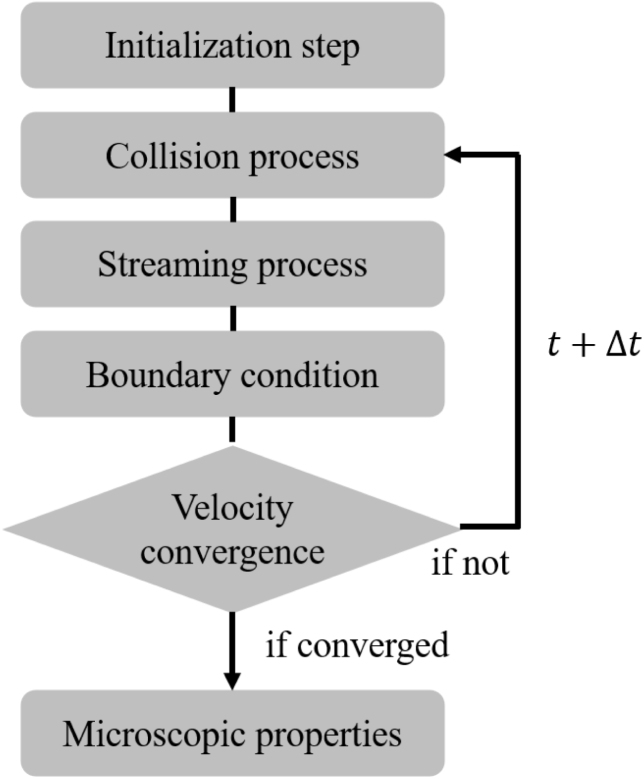
Flow-chart of the lattice Boltzmann implementation.

For this study, an in-house code had been developed further using the C/C++ programming language and parallelized with the compute unified device architecture (CUDA) to speed up the calculation on GPUs. The implementation workflow is presented in Fig. 4. The size of the computational domains, Reynolds number (Re) or external force (F), relaxation time (τ) were set by the user as input parameters, and the BCs will be discussed separately for each case in the later simulations. Specifically, the Reynolds number represents the dimensionless ratio of inertial force to viscous force, defined as Re=ρuL/μ and involving characteristic density ρ, velocity u, length-scale L and dynamic viscosity μ.

In addition to the fundamental steps outlined above, convergence was evaluated using the L2-norm to ensure that the simulation had reached a steady-state solution. This was measured by checking whether the relative difference between the velocity field in the current iteration and in the previous iteration was smaller than a user-defined convergence criterion according to Eq. (16). The iterative procedure would be repeated until this difference was below the desired threshold, set to 10^−10^ for all simulations in this work.

(16) $$E_{L2}=\sqrt{\frac{\underset{i,j}{\sum }{\left(u\left(x,t\right)-u\left(x,t-\Delta t\right)\right)}^{2}}{\underset{i,j}{\sum }u{\left(x,t\right)}^{2}}}.$$

The relative error based on the L2-norm was also used to check the accuracy of our numerical results whenever a comparison is possible. This is done by computing the difference between the results of our simulation (written $u^{num}$) and either the analytical solution $\left(u^{ana}\right)$ — as done in Eq. (17) — or published experimental values $\left(u^{exp}\right)$.

(17) $$E_{L2}=\sqrt{\frac{\underset{i,j}{\sum }{\left(u^{num}-u^{ana}\right)}^{2}}{\underset{i,j}{\sum }{\left(u^{ana}\right)}^{2}}}.$$

#### 2.4.3 Darcy’s law and unit conversion

After finishing a simulation, the permeability was calculated based on the obtained fluid velocity according to Darcy’s law, which relates the pressure gradient ΔP/L with the volumetric flux q as follows:

(18) $$q=-\frac{k}{\mu }\frac{\Delta P}{L},$$

where k is the (intrinsic) permeability and μ is the dynamic viscosity of the fluid. Since Darcy’s law is only valid when the Reynolds number is less than 1, the driving force in our simulation is set low enough to make the inertial term negligible, ensuring the validity of this condition. Accordingly, the permeability in LBM unit can be written as:

(19) $$k=-\frac{\rho U_{d}\nu }{\frac{\partial P}{\partial x}}$$

where $U_{d}$ denotes the Darcy’s volume-averaged velocity, ν denotes the kinematic viscosity, and $\frac{\partial P}{\partial x}$ denotes the pressure gradient in flow direction.

It should be noted that, in this study, the physical quantities obtained from the LB simulations are initially expressed in lattice units. To ensure that the final results carry physical meaning, the simulation outputs are converted from lattice units to the International System of Units (SI) based on the discrete time step (Δt) and lattice spacing (Δx).

To carry out this conversion, the “mlt” conversion framework as described by Gharibi et al. (2024b) is adopted. This framework is based on the fundamental dimensions of mass (m), length (l), and time (t). All physical quantities can be expressed by the following relation:

(20) $$\xi_{\mathrm{SI}}\left(m^{a}l^{b}t^{c}\right)=\xi_{l}\left[{\left(\rho {\left(\Delta x\right)}^{3}\right)}^{a}\Delta x^{b}\Delta t^{c}\right]\left(m_{l}^{a}l_{l}^{b}t_{l}^{c}\right)$$

where the subscript l and SI denotes lattice units and SI units, respectively, while a, b, and c represent the exponents corresponding to the units of a particular property (ξ). The specific relation between macroscopic quantities from lattice units to SI units are summarized in Table 2.

**Table 2:** Conversion between lattice unit and SI units.

Physical quantity	Unit conversion
Length (m)	$l_{\mathrm{SI}}=l_{l}\;\Delta x$
Time (s)	$t_{\mathrm{SI}}=t_{l}\;\Delta t$
Velocity ($m\;s^{-1}$)	$u_{\mathrm{SI}}=u_{l}\frac{\Delta x}{\Delta t}$
Kinematic viscosity ($m^{2}\;s^{-1}$)	$\nu_{\mathrm{SI}}=\nu_{l}\frac{{\left(\Delta x\right)}^{2}}{\Delta t}$
Permeability ($m^{2}$)	$\kappa_{\mathrm{SI}}=\kappa_{l}{\left(\Delta x\right)}^{2}$

## 3 Model validation

In this section, three benchmarks were systematically implemented step by step, to ensure that our in-house code had sufficient capability to accurately simulate 2D single-phase flow in real bread microstructures. In a first step, we strongly simplified the pore structure of bread as a collection of straight tubes filled with a pure fluid. For this scenario, a plane Poiseuille flow was simulated in Section 3.1 to verify the fundamental implementation of the LB model. Afterwards, solid obstacles were introduced in the geometry to represent the solid walls of a porous medium. This more complex model would be verified by the second benchmark in Section 3.2, which involved the creeping flow in a regular-shaped structure. Finally, in the third benchmark (Section 3.3), a real micro-CT image from the literature was used to evaluate the performance and accuracy of our model for a more realistic and irregular geometry leading to complex flow conditions. This completed the successful validation of our simulation model. All these simulations considered incompressible, Newtonian, single-phase, steady-state, and laminar flow conditions. Unless stated otherwise, all parameters are expressed in terms of lattice units (lu).

### 3.1 Plane Poiseuille flow

**Fig. 5:**
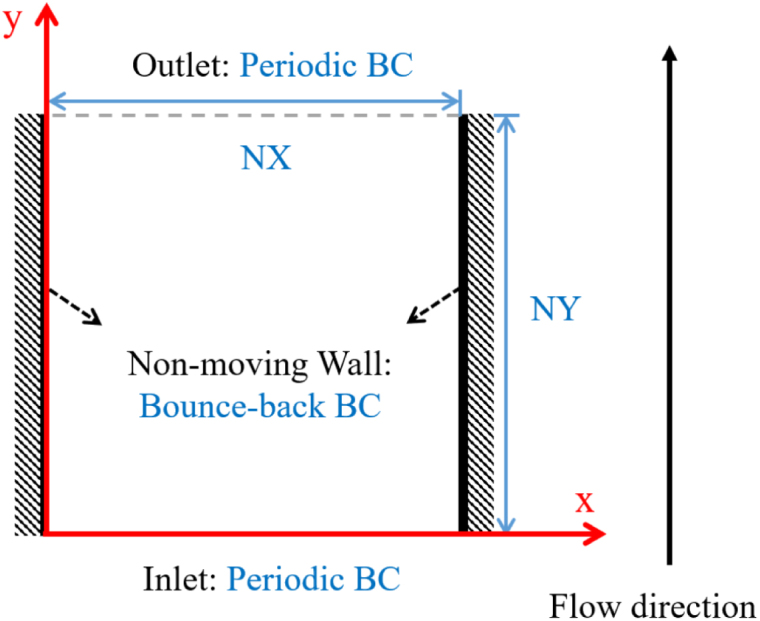
Schematic diagram of the 2D plane Poiseuille flow.

The plane Poiseuille flow is commonly used to verify the reliability and accuracy of numerical flow models. It was selected here as the first test case to check whether the flow behavior inside a channel predicted by our LB solver was correct. A sketch of the employed geometry is shown in Fig. 5. The computational domain was defined as a two-dimensional rectangular channel with length L and height H, discretized into NX and NY grid points along the x- and y-directions, respectively. The flow was driven along the y-direction, and the steady-state profile was obtained for laminar conditions. Here, ρ was set to 1 in lu. Assuming a creeping flow, the Reynolds number was set to 1 in this simulation. In addition, periodic BCs were applied at the inlet and outlet of the channel to ensure flow continuity, while bounce-back BCs were implemented on the left and right walls to enforce the no-slip condition for stationary walls. An external force field was applied to the domain as an external pressure gradient in order to drive the flow.

Beyond the numerical simulation using the LB method, an analytical solution derived from the Navier–Stokes equations is known as shown in Eq. (21). The accuracy of the LB simulation was verified by comparing these two results at steady-state.

(21) $$u_{y}=4u_{max}\frac{x\left(L-x\right)}{L^{2}}$$

Here, $u_{y}$ refers to the velocity in y-direction, $u_{max}$ refers to the maximum velocity, which occurs at the center of the channel, and x denotes the horizontal position along the channel.

**Fig. 6:**
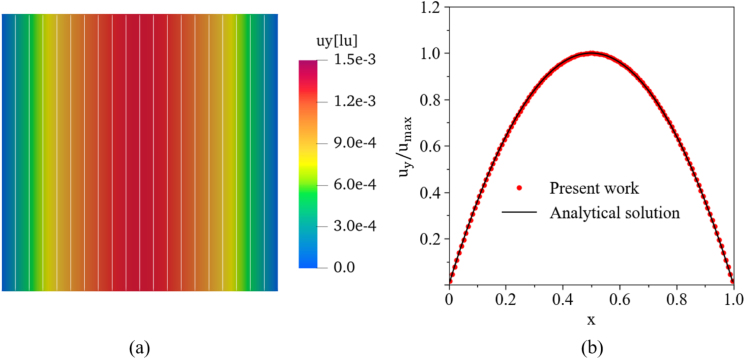
(a) Velocity field (colors) and streamlines (white lines) obtained by LBM. (b) Comparison of numerical velocity profile (symbols) and analytical solution (line).

The correctness of our simulation model could be verified by looking at Fig. 6(b), which showed an excellent agreement between LBM and analytical solution. The L2 norm of the relative error between these two results was only $3.93\times 10^{-6}$, demonstrating the high accuracy of our simulation. The standard parabolic velocity profile was found as expected, with straight streamlines parallel to the walls (Fig. 6(a)).

### 3.2 Flow in a homogeneous porous structure

**Fig. 7:**
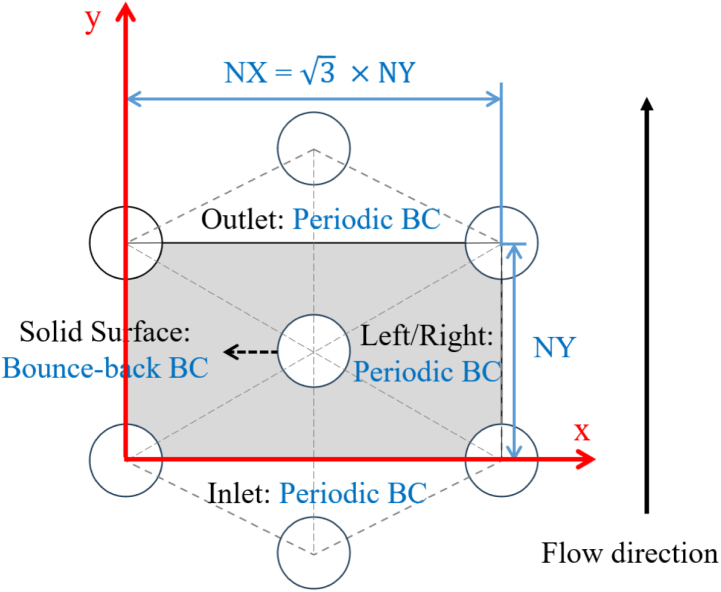
Schematic diagram of the 2D hexagonal obstacle geometry. The gray color identifies the really-employed simulation domain with corresponding boundary conditions.

To verify the accuracy of our LBM solver in a more complex case, a creeping flow in a 2D regular hexagonal structure was simulated. A sketch of the geometry is depicted in Fig. 7. It consists of a uniform hexagonal arrangement of five solid particles within a channel: four at the corners and one in the center. The particles have the same diameter, which were varied from 40 to 110 lattice units (lu) to adapt the porosity of the porous medium from approximately 0.2 to 0.9. The other simulation parameters were as follows: domain size = 440 (NX) × 254 (NY), external force $F=10^{-9}$, and relaxation time τ=1. Periodic BCs were applied at the inlet/outlet and left/right boundaries to get an infinitely large domain, while bounce-back BCs were imposed at the fluid-solid interfaces. The convergence of the simulation was monitored based on the average velocity.

**Fig. 8:**
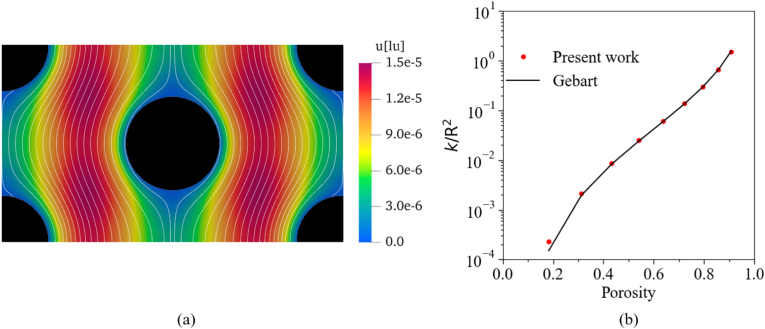
(a) Velocity field (colors) and streamlines (white lines) obtained by LBM.(b) Comparison of the numerically predicted permeability (symbols) with the analytical solution (line, from Gebart (1992)).

Fig. 8 presents the results of the LBM simulation. The velocity field (colors) and streamlines (white lines) in the fluid domain are shown in Fig. 8(a) for a particle radius of 60 lu, highlighting the preferential flow paths. The black regions represent the solid areas. The variation of permeability with porosity was calculated from these simulations by varying particle diameter, and the corresponding relationship is illustrated in Fig. 8(b). Here, the results are compared with Eq. (22), derived from Gebart’s study (Gebart, 1992), which provides a relationship between porosity and permeability in this case:

(22) $$\frac{k}{R^{2}}=\frac{16}{9\pi \sqrt{6}}{\left(\sqrt{\frac{\pi }{2\sqrt{3}\left(1-\phi \right)}}-1\right)}^{5/2},$$

where R represents the radius of the solid particles and ϕ represents the corresponding porosity of the porous medium. It is visible that the numerical predictions closely follow Gebart’s results, with a perfect agreement at high porosity that corresponds to the case of our target configuration — bread.

### 3.3 Flow in a heterogeneous porous structure

**Fig. 9:**
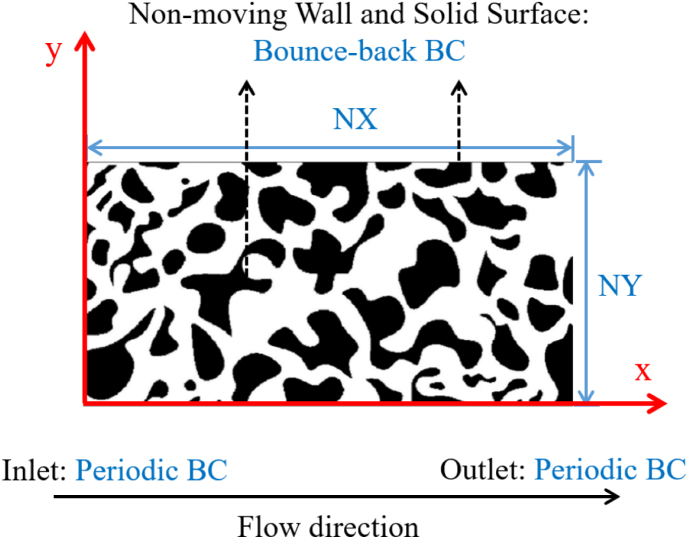
2D CT scan of a realistic heterogeneous geometry (Keller et al., 1997).

A porous structure obtained from digital CT scans was examined in the LB simulations in order to check the accuracy of our method for a realistic, heterogeneous geometry. The corresponding digital image (Keller et al., 1997), shown in Fig. 9, features a porosity of ϕ=0.567. The permeability of this structure based on numerical simulations was reported to be $k=3.990\times 10^{-8}$ m${}^{2}$ in Ju et al. (2019) but as $1.667\times 10^{-7}$ m${}^{2}$ in a more recent work by Ju et al. (2022). In the absence of any measurement or ground-truth solution for this case, these two values have been kept for comparison with our own numerical results using a domain size of 1800 (NX) × 888 (NY) lattice units, an external force of $F=10^{-10}$ and a relaxation time of τ = 1. Periodic BCs were applied at the left (i.e., inlet) and right (i.e., outlet) boundaries, as one of the most well-known types of open boundary conditions that have been extensively employed to simulate fluid flow in porous media (Hosa et al., 2016, Ho et al., 2021). They are obviously correct under the assumption of an infinite system, with the advantage of minimizing the influence of parameter variations (Babamahmoudi et al., 2023). The application of periodic BCs is equivalent to a physical structure where an infinite number of identical porous structures are connected in a chain-like arrangement, forming a continuous, closed-loop configuration. The bounce-back BCs were imposed at the top and bottom stationary walls, consistent with the approach used in the two referenced studies by Ju et al., 2019, Ju et al., 2022, as well as at all fluid-solid interfaces. Again, the convergence of the simulation was quantified based on average velocity.

**Fig. 10:**
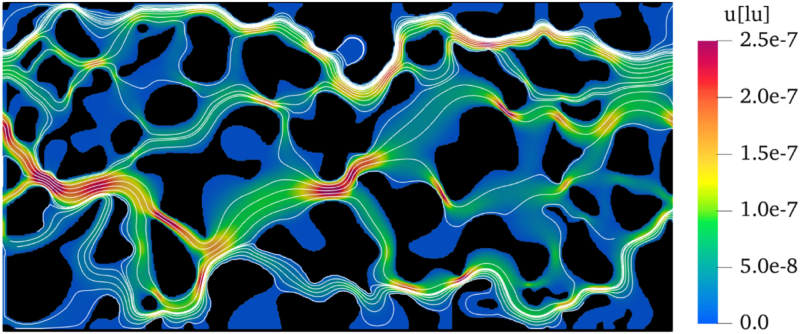
Velocity field (colors) and streamlines (white lines) obtained by LBM.

The permeability calculated based on our LBM simulation is $5.52\times 10^{-8}$ m${}^{2}$, which falls in-between the values mentioned in the two reference studies (Ju et al., 2019, Ju et al., 2022). This confirms the ability of our model to handle real heterogeneous structures. The streamlines and velocity field shown in Fig. 10 reveal the preferred pathways for the flow in this complex geometry, in particular important constrictions leading to a strong local acceleration. This intricate flow pattern arises from the heterogeneity of the porous structure, in which pore size distribution, connectivity, and tortuosity interact to control fluid dynamics. This highlights the challenges associated with predicting flow patterns in real porous media.

### 3.4 Flow in a FCC structure

**Fig. 11:**
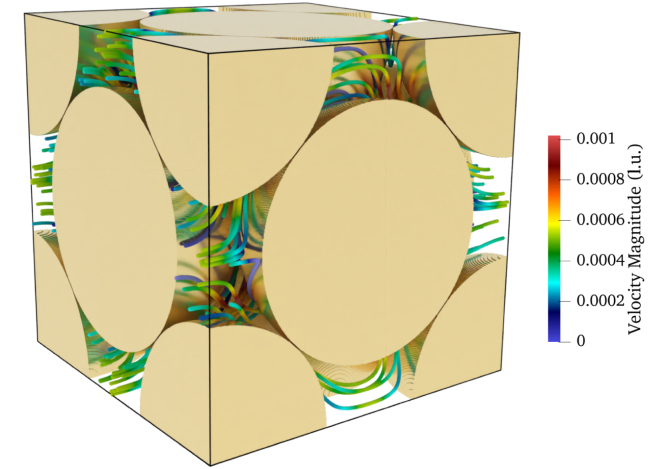
Geometry of the face-centered cubic (FCC) porous structure and corresponding streamlines of the simulated fluid flow within the domain (domain size: $\sqrt{2}D\times \sqrt{2}D\times \sqrt{2}D$).

To demonstrate the accuracy of our method in 3D cases, a face-centered cubic (FCC) structure was examined. The geometry, shown in Fig. 11, is composed of spheres with identical diameter D, where D is related to the cube side length L=NX=NY=NZ by $D=L/\sqrt{2}$. The spheres form the solid phase and are located at the cube corners and face centers, while the remaining space constitutes the fluid region, yielding a porosity of 0.25952. Meanwhile, to investigate the influence of spatial resolution on the accuracy of our method, LB simulations were conducted at different domain sizes (32, 64, 128, 256, and 512 lattice units) under the same porosity. Creeping-flow conditions were ensured by applying a uniform body force of $F=10^{-7}$ (l.u.) in all cases. The corresponding Reynold number and other parameters for each case are listed in Table 3. In addition, the relaxation time τ was set as 0.6 for all cases considered in this section.

The permeability of the FCC structure was determined by solving the unsteady Stokes equations by Chapman and Higdon (1992). The reported dimensionless permeability, $k/D^{2}=1.7360\times 10^{-4}$, is adopted here as the reference value. Table 3 summarizes the permeabilities calculated from our simulation results at different domain sizes, corresponding to different spatial resolutions. It is clear that as the resolution increases, the calculated permeability gets closer to the reference one; especially when the domain sizes increase to 256 and 512, the difference is smaller than 1%. Fig. 11 presents selected streamline within the FCC geometry. All these simulations were performed on the Sofja high-performance computing (HPC) system using a single NVIDIA A100 80 GB GPU. The corresponding computation times are provided in Table 3 for reference.

**Table 3:** Resolution study of normalized permeability and computational performance.

L	Re	$k/D^{2}\times 10^{4}$	Error (%)	Wall-clock (s)	Iterations	MLOPS	Memory (GB)
32	$1.94\times 10^{-4}$	2.042	17.62	1	3000	98.3	0.43
48	$6.40\times 10^{-4}$	1.937	11.56	2	3000	165.9	0.47
64	$1.49\times 10^{-3}$	1.872	7.82	4	4000	262.1	0.52
128	$1.15\times 10^{-2}$	1.768	1.84	51	10 000	411.2	1.27
256	$9.19\times 10^{-2}$	1.742	0.35	1126	33 000	491.7	7.29
512	$7.37\times 10^{-1}$	1.737	0.03	31 146	117 000	504.2	55.41

## 4 Results and discussion

It is now possible to use our fully-validated LBM solver for simulating flows in real porous media, particularly liquid flow through the 2D processed bread images. With structural properties such as anisotropy, porosity, connectivity, pore size distribution (PSD), and tortuosity already characterized from these images, the influence of these structural properties on flow behavior and permeability can be systematically explored.

**Fig. 12:**
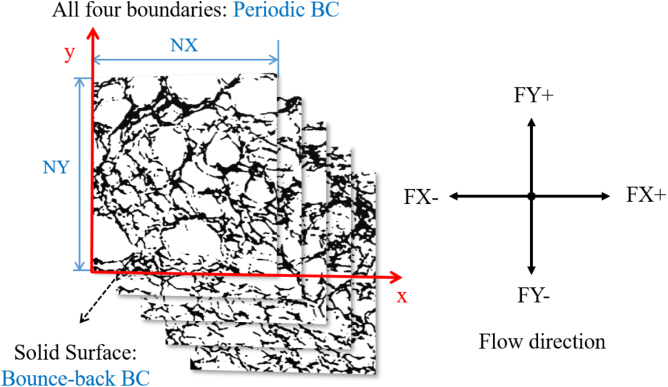
Schematic diagram of 2D bread geometry showing five slices corresponding to the different porosity ranges P65, P67, P69, P71, P73.

In the simulation, the four surrounding boundaries were set as periodic BCs, following the approach of Meng et al. (2020) for permeability measurements, ensuring that fluid and solid grid interactions occur without interference from boundary effects. All contact points between fluid and solid surface were set to bounce-back BCs, as shown in Fig. 12. The remaining parameters were as follows: $F=10^{-5}$, τ = 1.

**Fig. 13:**
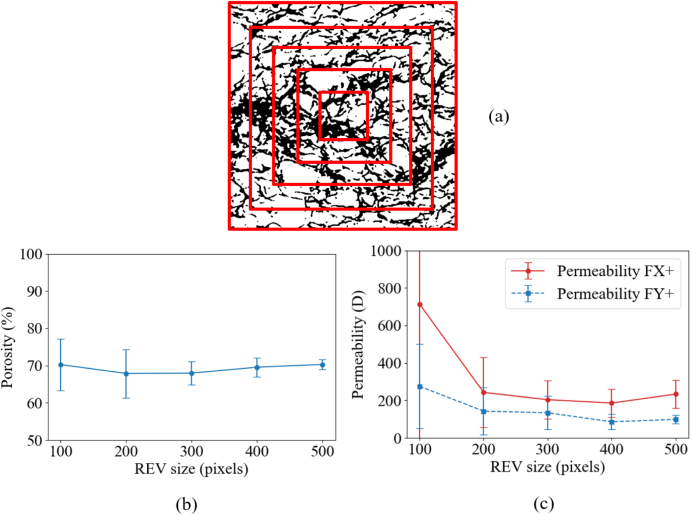
(a) Selection strategy of representative elementary volume (REV) regions within the bread microstructure image. (b) Variation of porosity with increasing REV size. (c) Variation of permeability in the X and Y directions with increasing REV size.

In addition, representative elementary volume (REV) analyses were conducted to determine the image size that can represent the bread microstructure, based on the convergence behavior of both porosity and permeability. Five representative bread samples, covering a porosity range of 65% to 73%, were used for the 2D evaluation, and the corresponding selection strategy and results are presented in Fig. 13. It can be observed that when the domain size exceeds approximately 300 × 300 pixels, both porosity and permeability become stable with only negligible variations. Based on this observation, and considering computational efficiency, an image size of 400 × 400 pixels was selected as the REV for the subsequent 2D simulations, corresponding to a computational domain of 400(NX)×400(NY).

**Fig. 14:**
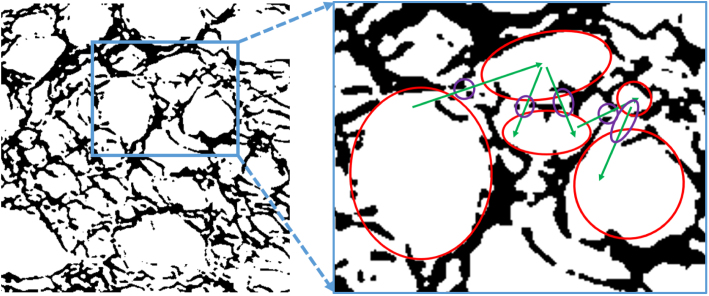
Selected details of the pore structure of bread. The red figure is a zoom on the blue rectangle shown in the left image.

### 4.1 Characteristics of bread’s pores

Through the analysis of CT images, as shown in Fig. 14, it is observed that the flow paths inside the bread involve two very different kinds of structures. First, the main pores, resulting from the incorporation of bubbles during dough mixing and yeast fermentation, as well as bubble expansion during baking, are highlighted by red circles in the right part of Fig. 14. But also numerous small broken holes are found along the cell walls, resulting from the breakage of bubbles during baking, some of them being exemplarily indicated by purple circles in the right part of Fig. 14. These smaller passages connect the larger pores together and lead to the open structure of the bread, as observed in the studies by Wang et al. (2011) and Chakrabarti-Bell et al. (2014). As explained by Wang et al. (2011), the presence of airflow channels extending throughout the tested bread was confirmed by blowing air into one side of the slice and feeling the air exit from the other side.

### 4.2 Analyzing bread anisotropy

Anisotropy arises because of the irregular shape, size, and random distribution of pores and small broken holes within the crumb (Laurent et al., 2008). To investigate the anisotropy of our bread structure, the same flow was simulated along four different directions (refer to Fig. 12): along the x-axis (denoted as FX+, representing flow from left to right, or FX-, representing flow from right to left), and along the y-axis (denoted as FY+, representing flow from bottom to top, and FY-, representing flow from top to bottom).

**Fig. 15:**
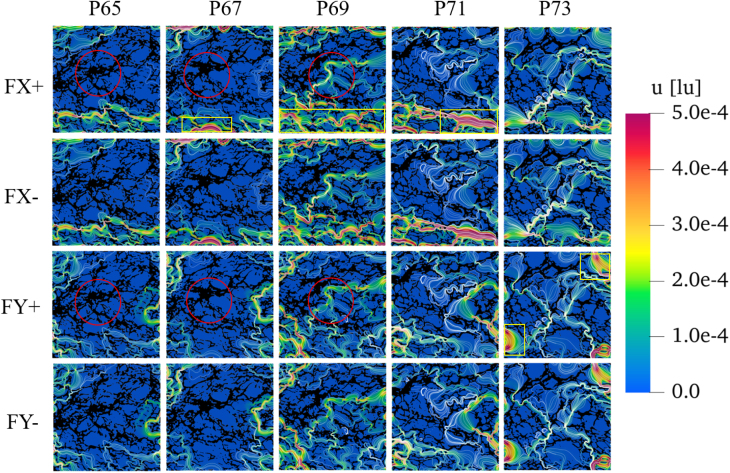
Velocity field (colors) and streamlines (white lines) in the bread microstructure for different flow directions. From top to bottom: FX+ (from left to right), FX- (from right to left), FY+ (from bottom to top), FY- (from top to bottom). The five columns (from left to right) correspond to increasing porosity, from 65% to 73%. The porosity value is indicated after the P appearing on top of the corresponding column. The color scale is kept identical for all figures.

Fig. 15 shows the velocity fields and streamlines (white lines, best seen by enlarging the pictures) obtained in these four simulations, with colors representing velocity magnitude (red indicating high velocities and blue indicating lower ones). The black areas highlight the solid microstructures of the bread. By comparing the two top lines it can be observed that, in the x-direction, the streamlines and preferred flow paths are the same for both FX+ and FX- (from left to right or from right to left). The same behavior can also be seen for the FY+ and FY- directions comparing the two bottom lines of the figure. This indicates reversibility of the flow. In contrast, the flow behavior in the horizontal and vertical directions (comparing the two top lines to the two bottom lines in Fig. 15) differs significantly, with distinct preferred flow paths (and also permeability values, as reported later in Fig. 17), indicating a strong anisotropy of the considered bread structure. This remains true for the whole range of porosities relevant for this material (porosity increasing from left to right in Fig. 15, as indicated by the P-value appearing on top of the corresponding column).

**Fig. 16:**
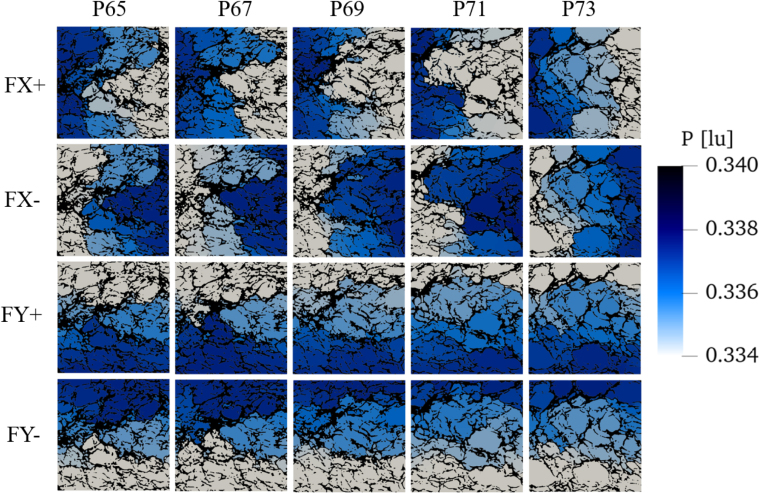
Pressure distribution in the bread microstructure for different flow directions. See Fig. 15 for further details.

Fig. 16 shows the associated pressure distribution with colors (from gray, low pressure, to dark blue, high pressure). The black areas highlight the solid part of the bread’s microstructure. As expected, the fluid pressure decreases gradually with increasing distance from the inflow boundary, indicating pressure loss, a trend that is also consistent with the findings in the literature, e.g. Sato et al. (2019). However, the presence of small throats significantly impacts the local pressure drop. For this reason, the pressure becomes progressively non-uniform across different parts of the structure. Regions with more interconnected or wider pores exhibit smoother pressure changes, whereas areas dominated by narrow throats and disconnected voids lead to a higher pressure drop, which emphasizes the importance of pore and throat size and their distribution.

The mirrored variations of pressure for FX+ and FX-, as well as for FY+ and FY-, further confirm our previous results regarding reversibility and anisotropy of the fluid flow. This indicates that a single simulation is sufficient to analyze the flow in one specific direction. Consequently, the rest of this article focuses solely on the FX+ and FY+ simulations, which is sufficient to describe anisotropy.

### 4.3 Analysis of permeability

The relationship between porosity and permeability in bread remains uncertain. In order to investigate possible variations in permeability at a fixed porosity level, three different 2D slices were selected for each porosity group. As described in Section 2.3, we first analyzed the porosity distribution of all slices along the Z-direction and selected images from positions lying sufficiently far away (to avoid structural similarity caused by local clustering). Within each group, slices with porosity values close to the target range (meaning, e.g., between 0.670 and 0.672 for P67) were chosen to quantify the influence of topological variations on the results. These representative slices, which exhibit similar porosity but different microstructural features, were then used for flow simulations, with corresponding results summarized in Table 4. In this manner, it is possible to check corresponding variations and derive statistics. Table 4 presents the permeability (k) calculated using our LBM simulations for all these cases, along with two basic structural properties of the corresponding bread slice, porosity and effective porosity (EP). While in most established correlations from the literature, permeability depends only on porosity, the results in this table reveal that, even for the same porosity, permeability can vary significantly due to details of the structure. For the same case, for instance, P69 with a porosity of roughly 69%, the computed permeability varies in x-direction by 47%, in y-direction by 57%. The small variations in effective porosity (less than 4% for this particular case) cannot explain these very large differences.

Following this unexpected observation, it was decided to examine separately the effects of various structural parameters on the permeability of bread. To simplify the analysis, in the rest of this study, only the first sample (corresponding to the first line in Table 4) from each of the five porosity levels was considered. Detailed flow information was then integrated to systematically investigate the effects of various parameters on the internal flow behavior within the corresponding bread sample.

**Table 4:** Relationship between (effective) porosity measured with μCT and permeability computed by LB simulation in x and y directions.

Case	Porosity	Effective porosity	k in x-dir.	k in y-dir.
	%	%	(in Darcy, D)	(in Darcy, D)
P65	0.653	0.598	117.4	51.9
	0.656	0.584	192.6	86.7
	0.657	0.535	194.9	61.2

P67	0.670	0.649	151.4	59.6
	0.671	0.591	198.2	106.3
	0.672	0.650	132.6	77.6

P69	0.691	0.660	292.7	130.6
	0.692	0.638	156.1	56.3
	0.692	0.645	162.9	79.2

P71	0.712	0.682	276.8	95.8
	0.712	0.672	379.7	178.0
	0.712	0.692	376.1	76.4

P73	0.731	0.668	176.9	145.3
	0.730	0.696	274.2	70.4
	0.732	0.688	94.8	81.5

#### 4.3.1 Effect of porosity on permeability

It is widely accepted that porosity is a key parameter in determining permeability. During bread manufacturing, porosity helps assess the degree of expansion and shrinkage during fermentation (Germishuys and Manley, 2021) and storage (Chen et al., 2021b, de Lima et al., 2023, Yu et al., 2023), as well as investigate the effect of freezing (Li et al., 2024).

**Fig. 17:**
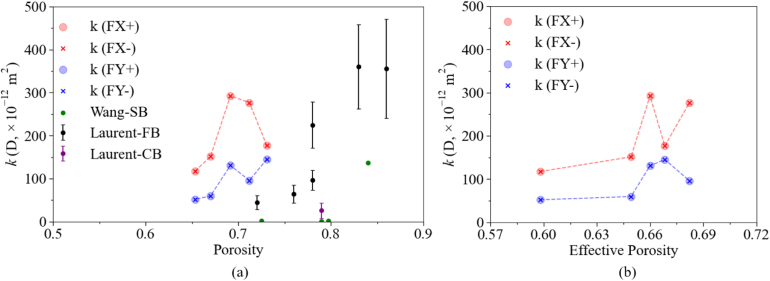
Variation of permeability (k) with porosity (left) and effective porosity (right) for bread structure, compared to results from the literature in subfigure (a). There, FB denotes French Bread, CB denotes Commercial Brioche, and SB denotes Sandwich bread.

Our results from CT images show that the chosen 2D slices of our bread sample have porosity values ranging from 0.65 to 0.73. These values are consistent with typical bread porosity values reported in studies such as Germishuys and Manley (2021) and Besbes et al. (2013) at micro-scale, as well as Li et al. (2022) at macro-scale. Fig. 17 shows the variations of permeability vs. porosity and effective porosity for the five representative samples introduced previously, one for each porosity class P65, P67, P69, P71, P73. The results indicate that the corresponding permeability values, ranging from 100 to 500 D, align well with experimental data reported in the literature, such as 45 ± 16 D to 361 ± 98 D by Laurent et al. (2008) and 0.28 D to 136.97 D by Wang et al. (2011). This consistency further confirms that our model effectively simulates fluid transfer in bread structures. It is worth noting that the permeability values obtained in this study are somewhat higher than those reported in previous studies. This may be attributed to differences in the type of bread used and variations in internal microstructure characteristics. Indeed, the permeability values reported for French bread (FB) (Laurent et al., 2008) exhibit large variations, suggesting that permeability is highly sensitive to structural variability, even within the same type of bread.

Fig. 17(a) reveals a general increase of permeability with porosity due to larger spaces available for fluid flow, as expected; all empirical/analytical porosity-permeability relationships are predominantly monotonically increasing functions of porosity. However, our results do not show such a clear monotonic trend, with local drops both in the x-direction and in the y-direction. This behavior might be attributed to the presence of isolated closed pores, which increase porosity but do not contribute to permeability. To address this, the effective porosity for each sample was calculated, and the variation in permeability based on it was plotted in Fig. 17(b).

Even these calculations indicate that the magnitude of permeability does not exhibit a monotonic increase with increasing effective porosity. For instance, in x-direction, the maximum permeability is observed at an effective porosity of 0.66 before decreasing again. This finding suggests that permeability is not solely dependent on (effective) porosity but is also strongly influenced by other factors such as pore size, shape, and connectivity (Laurent et al., 2008). Ignoring these factors oversimplifies the complex structure of the bread. Therefore, it was decided to analyze further the influence of connectivity, pore size distribution, and tortuosity on the permeability of bread.

#### 4.3.2 Effect of connectivity on permeability

**Fig. 18:**
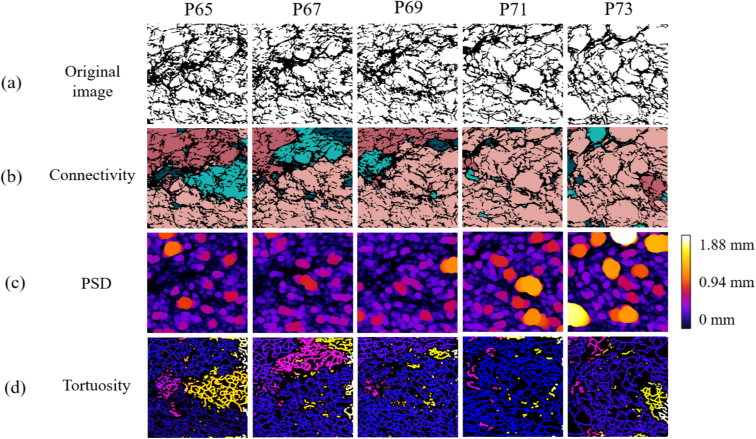
Structural analysis of bread CT scans.

This section focuses on the critical role of global connectivity of the available pore space, determining the possible pathways for fluid movement and the potential constraints on flow direction. Connectivity of pores, as a topological factor, is crucial for fluid movement within bread (Aghajanzadeh et al., 2024). To study the pathways by which fluid can pass through the internal bread structure, these connected groups of pores were labeled by analyzing numerically the CT images discussed previously. Fig. 18(b) provides a colored representation of groups of connected pores. In this figure, the different possible pathways are colored differently to help visualize and understand how the pore structure in the bread facilitates or limits the transport of fluids.

From the results in Fig. 18(b), it is observed that, for the P65, P67, and P69 cases, there is no continuous color along the y-axis, indicating a lack of connected pathways in this direction. This strongly limits any vertical fluid flow between the top and bottom sections, thereby leading to low permeability in both the P65 and P67 cases. Additionally, the fluid movement observed in the central region is only due to the periodic BCs, which allow the fluid to enter through the left and right boundaries. In contrast, along the x-axis, continuous pathways are present, connecting the left and right boundaries. For instance, the red region in P65 spans the full width of the image, facilitating horizontal fluid flows. However, for cases P71 and P73, the pink region dominate most of the image, indicating that the pore networks are continuous along both directions and facilitate fluid flow throughout the entire structure. The maze-like structure of bread was also discussed in Wang et al. (2011). In their case, all the open cells in bread were connected, creating a single, large, highly tortuous cell that was open to atmosphere.

#### 4.3.3 Effect of pore size distribution (PSD) on permeability

**Fig. 19:**
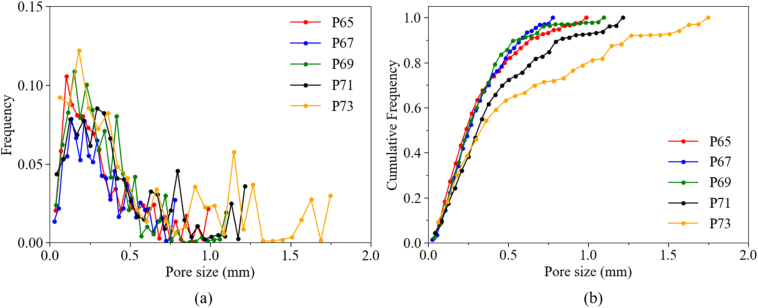
Frequency and corresponding cumulative frequency of pore size of bread micro-structure.

The non-uniform size and irregular distribution of pores contribute to the heterogeneous structure of the bread. Therefore, understanding the pore size distribution and its influence on permeability is crucial. Fig. 19 displays the frequency and corresponding cumulative frequency of pore size. Additionally, the pore size distribution map, which visualizes the spatial information regarding pore size, is shown in Fig. 18(c). It can be observed that the rising trend in cumulative frequency (Fig. 19(b)) of cases P65, P67 and P69 is almost the same, indicating similar proportions of small and larger pores, with a maximum size of 0.99mm, 0.78mm, and 1.10mm, respectively. The P71 and P73 cases, however, have significantly more and larger pores, with maximum sizes of 1.22mm and 1.75mm. This distinction is further evidenced in the spatial distribution maps, where these two cases display larger bright regions, signifying the presence of more extensive pores. The increased number of large pores (greater than 1.2mm) in the P71 and P73 cases also reflects a slightly higher degree of heterogeneity compared to the P65, P67, and P69 cases, consistent with previous findings (Laurent et al., 2008). Moreover, the pore size distribution curves (Fig. 19(a)) of all cases do not follow a Gaussian distribution, similar to the findings for bread reported by Besbes et al. (2013).

By focusing on the influence of pore size distribution on flow behavior, it is observed that fluid velocity increases when flowing from larger pores into smaller pores, accompanied by a rapid drop in pressure. Correspondingly, it can be observed that the color of the velocity magnitude changes from green to red in the velocity field (Fig. 15), and a sudden color shift (e.g., from dark blue to light blue) can be found in the pressure field (Fig. 16). This phenomenon is attributed to the bottleneck effect, where larger pores provide more space for fluid flow, resulting in less pressure loss, whereas smaller pores increase flow resistance and decrease permeability (Huang et al., 2020). Thus, it becomes clear that small-size pores, acting like valves, should be given special attention since they lead to a much higher flow resistance. Consequently, even the extended Carman–Kozeny’s (C-K) model, which is applicable to media such as soil or expanded solid foams, is not necessarily suitable for grain-based products (Laurent et al., 2008).

In fact, as mentioned earlier, the air pockets created by yeast fermentation are typically considered to lead to large pores, while the broken holes formed during baking are usually regarded as small pores. This means that the focus on small pores can be indirectly shifted to the broken holes. Following this argumentation, it is worth noting that both the size and location of broken holes are important. For example, the P67 and P69 cases in x-direction, which show connected small pores, as well as cases P71 (in x-direction) and P73 (in y-direction) cases, which show uniformly connected large pores, have greater permeability than all other conditions. Yellow squares highlight corresponding regions in Fig. 13, suggesting that: uniform large-size holes can effectively mitigate the effect of narrow throats, ensuring that as much fluid as possible flows through; while a uniform distribution of small-sized holes can also help maintain a consistently high flow rate. Additionally, it is also important to know the local connectivity of the pores, which was measured by tortuosity and branching, as discussed next.

#### 4.3.4 Effect of tortuosity on permeability

Tortuosity quantifies the straightness of the paths within the bread structure and is defined as the ratio between the distance of actual flow paths and the corresponding straight-line connection (Gao et al., 2018).

Firstly, to better visualize the fluid flow paths, the channels have been skeletonized, as shown in Fig. 18(d). The different colors are used to distinguish individually connected regions, consistent with the connectivity map shown in Fig. 18(b). Based on this representation, we calculated the average tortuosity of all the paths and identified branch points where three or four paths converge, referred to as triple points and quadruple points, respectively. The results of these calculations are displayed in Table 5.

**Table 5:** Tortuosity and branch points of bread micro-structure.

Case	Tortuosity	Triple points	Quadruple points
P65	1.157	495	42
P67	1.164	539	47
P69	1.159	577	64
P71	1.172	447	33
P73	1.173	426	32

A lower tortuosity indicates that the flow path is closer to a straight line, reducing losses and allowing easier fluid flow. More branches, represented by a higher number of triple and quadruple points, provide more alternative paths for fluid movement. Both features can improve fluid transport within the bread structure. The results for the P69 case, which has the highest permeability in the x-direction (292.7 D), clearly demonstrate this point. Additionally, compared to the 495 (resp. 539) triple points and 42 (resp. 47) quadruple points in the P65 (resp. P67) case, Case P69 comes with a significantly higher number of triple points (577) and quadruple points (64). These additional branches facilitate fluid transport through the central region, as shown in the corresponding red circles in Fig. 15, therefore increasing permeability, confirming our earlier hypothesis from Section 4.3.2. Also compared to the P71 and P73 cases, a lower tortuosity and more branches are found in P69, as shown by the denser branching network in Fig. 18(d). Those facilitate fluid transport, resulting in greater permeability.

However, it should be noted that the roles of tortuosity and branch points cannot be considered separately but should be analyzed together with other structural characteristics. For instance, in the same CT slice, tortuosity and the number of branch points remain unchanged across different flow directions, whereas the corresponding permeability varies, especially with large differences observed in cases P69 and P71. In the P69 case, the y-direction lacks connectivity compared with the x-direction; thus, although the overall tortuosity is relatively low and many branches are present, the absence of percolative pathways prevents efficient flow, leading to reduced permeability. In the P71 case, the effect of tortuosity and branch points is less pronounced in either direction; instead, the relatively higher permeability along the x-direction is primarily attributable to the presence of a dominant high-flow channel.

### 4.4 Spearman correlation analysis

To further quantify the influence of structural characteristics on permeability, a Spearman correlation analysis was conducted, based on the simulations performed on 100 2D CT images of bread. The analysis considered a set of structural descriptors, including total porosity and effective porosity, binary indicators of connectivity, statistical measures from the pore size distribution (D10, D50, and D90), tortuosity, as well as the number of triple points and quadruple points. Specifically, the main connectivity and secondary connectivity, referring to the continuity of the pore network in the direction of the applied force and the corresponding perpendicular direction, were defined based on the Section 4.3.2. These two indicators were encoded as binary variables: 1 indicates the presence of a continuous connected path across the domain in the specified direction, and 0 indicates the absence of such a path. Meanwhile, three commonly used parameters: D10, D50, and D90, were selected for the characterization of the pore size distribution (PSD), indicating that 10%, 50%, and 90% of the pores have diameters smaller than the respective values (Gaol et al., 2020).

For each parameter, the Spearman correlation coefficient ($r_{s}$) and its corresponding p-value were calculated to evaluate the strength and statistical significance of the correlation with permeability. The coefficient $r_{s}$ ranges from −1 to 1, where positive values indicate a monotonic increasing relationship and negative values indicate a decreasing one. To distinguish different levels of statistical significance, p-values were measured using asterisks: ∗ for p<0.05, ∗∗ for p<0.01, and ∗∗∗ for p<0.001. Among them, p<0.05 was selected as the threshold for statistical significance at the 95% confidence level, indicating that the observed correlation is statistically meaningful.

**Fig. 20:**
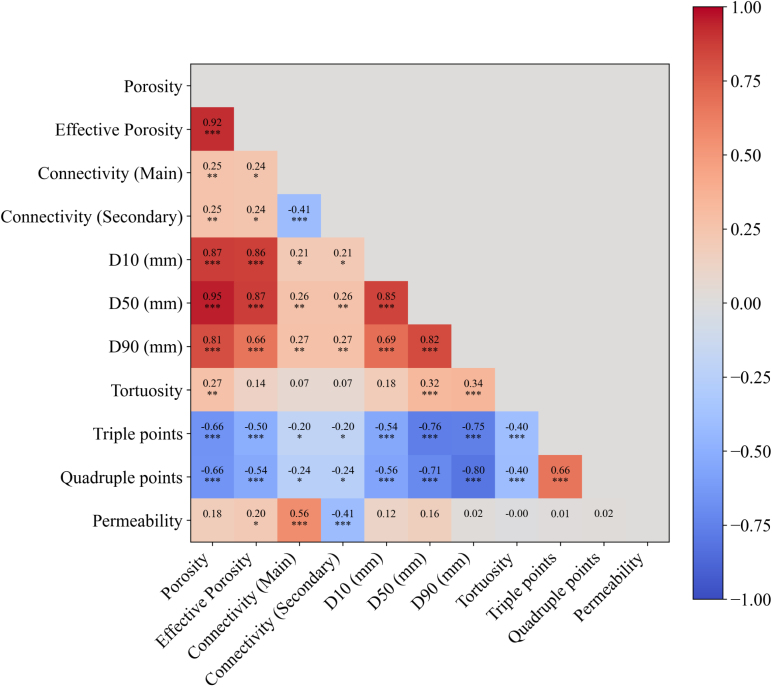
Correlation coefficient distribution diagram of parameters. Values indicate Spearman correlation coefficients. Statistical significance is denoted by asterisks: ∗ for p<0.05, ∗∗ for p<0.01, and ∗∗∗ for p<0.001.

Fig. 20 shows the results of the Spearman correlation analysis between permeability and the various geometrical descriptors. Three parameters exhibit statistically significant correlations with permeability (p<0.05): effective porosity, main connectivity and secondary connectivity. Among them, secondary connectivity is negatively correlated with permeability, while the other two parameters exhibit positive correlations.

Main connectivity exhibits the strongest positive correlation with permeability ($r_{s}=0.56$), underscoring the critical role of pore network continuity along the primary flow direction. A well-connected structure facilitates more direct flow paths, thereby enhancing permeability, consistent with our previous findings. In contrast, secondary connectivity shows a moderate negative correlation with permeability, possibly due to flow diversion into non-primary directions, leading to more dispersed and less efficient transport. Notably, this effect of secondary connectivity is not adequately captured by average tortuosity, as indicated by the weak correlation between secondary connectivity and tortuosity ($r_{s}=0.07$). This is because tortuosity is averaged over all paths, including those with minimal flow, which also explains the absence of any correlation with permeability ($r_{s}\approx 0$). Both porosity and effective porosity show modest positive correlations with permeability ($r_{s}=0.18$ and 0.20, respectively), suggesting that pore volume friction alone, while providing some indication of the available flow space, does not sufficiently predict permeability without considering structural connectivity, which also supports our earlier conclusion.

Regarding the PSD parameters, as previously analyzed based on the image, the influence of large pores appears to be minimal — the correlation coefficient for $D_{90}$ is only 0.02. In contrast, $D_{10}$ and $D_{50}$ are more representative of pore and throat sizes. However, $D_{10}$ represents only the smallest 10% of pores, whereas $D_{50}$, as the median value, more comprehensively captures typical throat size and shows a stronger correlation with permeability. Indeed, as shown in the PSD curves (Fig. 19(b)), $D_{50}$ values in all cases are lower than half of the maximum pore size, suggesting that this relatively small value can serve as a representative measure of the constrictions that actually govern fluid flow. This finding is also consistent with earlier observations that well-connected small to medium pores can effectively enhance permeability.

The parameters related to tortuosity and branching show negligible correlations with permeability ($r_{s}<0.05$). Although an increased number of triple and quadruple points may theoretically provide more flow paths, they can also divert fluid away from the main channels, reducing overall flow efficiency. Similarly, the explanatory power of average tortuosity is limited, as it includes non-conductive or low-flow paths that do not accurately represent the actual flow behavior.

In summary, connectivity shows the strongest correlation with permeability in the bread microstructure, highlighting its fundamental role in controlling fluid transport within porous media. Porosity and effective porosity exhibit moderate positive correlations, suggesting that pore volume contributes to permeability when sufficient structural connectivity is present. Pore throats, acting as flow-controlling constrictions, thus reduce permeability. However, this restrictive effect may be partially offset in systems with well-connected pore networks that enable more efficient fluid passage. In contrast, average tortuosity and the number of branch points display negligible correlations with permeability. These results reflect the multi-factorial nature of pore structural control on permeability, with connectivity emerging as the most critical factor among the evaluated geometric descriptors.

Moreover, after systematically identifying the key geometrical descriptors governing bread permeability, establishing a quantitative relationship between these descriptors and permeability would further enhance the practical applicability of the present study. As a preliminary step, three regression models (linear–linear, log–linear, and log–log) were constructed to describe the relationship between permeability and the selected geometrical descriptors. However, all three models yielded relatively low coefficients of determination ($R^{2}<0.40$). Although general trends were captured, the predictive capability remained limited. Future work will focus on developing a mathematical predictive model, based on the findings of this study, that quantitatively links the extracted geometrical descriptors to permeability. Furthermore, deep-learning approaches will be employed to capture nonlinear relationships and improve predictive accuracy.

### 4.5 Towards 3D simulations

In the previous sections, the influence of local geometrical features on the velocity fields and pressure distributions was investigated. Taking advantage of the reduced computational cost of 2D simulations, statistical studies based on a large number of slices can be performed, revealing the underlying coupling between structure and flow. However, extending to the third spatial dimension may naturally introduce some differences in topology and — as a result — simulation details. While 3D simulations provide a closer representation of the real physical system, the very high costs of such studies preclude any systematic parameter variation. To further validate and extend our findings, and to assess the validity of the trends identified in 2D, it was decided to conduct a single simulation of the 3D configuration and to perform a first analysis of the resulting permeability values and directional anisotropy.

**Fig. 21:**
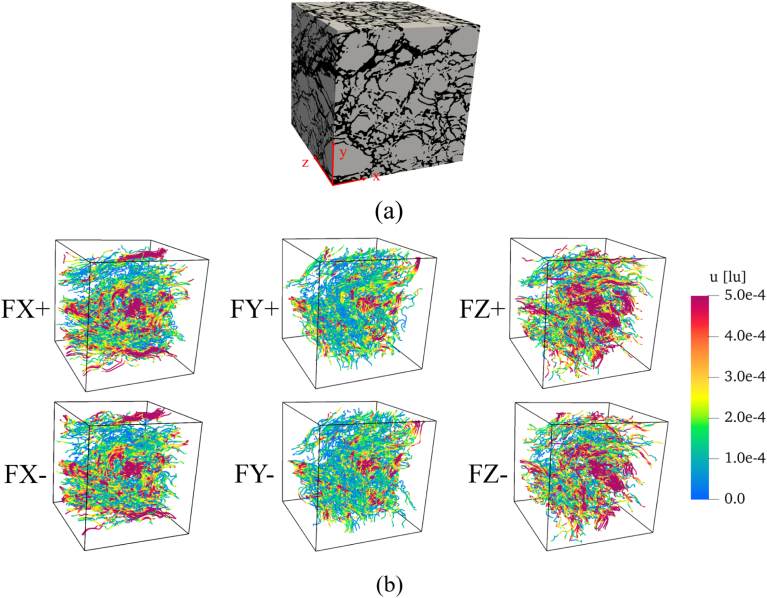
Simulated bread microstructure geometry and corresponding velocity fields under different flow directions. (a) Simulated bread microstructure geometry. (b) Velocity field (colored streamlines) within the bread microstructure along the x, y, and z directions.

Fig. 21(a) presents the geometry of the bread microstructure used in the 3D simulations, reconstructed by stacking the same 2D images employed previously using identical parameters. Before proceeding with the analysis, a preliminary REV assessment was performed on the single available 3D sample, using the same convergence criteria applied to the 2D analysis: porosity and permeability. The REV regions were selected following the same strategy as in 2D, with cubic subdomains extracted from the center of the sample and progressively expanded outward. The porosity and permeability values corresponding to different domain sizes are summarized in Table 6. It is indicated that porosity shows only small variations for all cases, while permeability shows a tendency toward stabilization once the domain size exceeds 300 × 300 × 300 voxels. Therefore, for the 3D case, the chosen volume (400 × 400 × 400 voxels) is considered sufficiently large to be used as a REV.

**Table 6:** Effect of REV size on porosity and directional permeability in 3D simulations.

REV size (voxels)	REV size (mm)	Porosity (%)	Permeability k (D)		
			FX+	FY+	FZ+
100	1.87	68.4	844.0	329.5	1002.3
200	3.74	69.4	618.3	306.3	1016.2
300	5.61	68.3	526.2	248.4	828.5
400	7.48	69.8	561.9	249.1	798.3

Fig. 21(b) illustrates the flow fields along the x-, y-, and z-directions, corresponding to the FX and FY groups discussed above, and to the third, perpendicular direction. The calculated permeability values for these six directional simulations are 561.9 D (FX+), 561.7 D (FX-), 249.1 D (FY+), 249.1 D(FY-), 798.3 D (FZ+), and 797.7 D (FZ-).

Although the permeability obtained from the 3D simulation is higher than that from the average of the 2D cases, the overall trends and directional anisotropy remain consistent. The permeability in the x-direction is larger than in the y-direction, in agreement with the 2D simulation results. Both the flow behavior and permeability patterns indicate that the bread microstructure exhibits strongly anisotropic flow properties, confirming the qualitative conclusions drawn from the 2D analysis. Specifically, the flow in the same direction shows nearly identical patterns and permeability values, regardless of whether the inlet and outlet are exchanged; while results in x, y and z direction differ strongly.

These comparisons indicate that the 3D simulations reveal the same directional anisotropy in flow behavior observed in the 2D cases. However, the high computational cost of 3D simulations limits the number of cases that can be performed, making systematic investigations with parameter variations currently impractical. In this context, 2D simulations are highly valuable for capturing qualitative trends at a comparatively low computational cost.

In addition, to assess the feasibility of using numerical coarsening to reduce computational cost and simulation time, the permeability obtained from the original image was compared with that computed from images at lower voxel resolutions generated through numerical coarsening. The numerical coarsening procedure followed the approach described in the study of Shah et al. (2016), in which a coarser grid was mapped onto the original lattice. Specifically, for each newly generated cell, the solid fraction was calculated based on its overlap with the original grid. A cell containing more than 50% solid was classified as solid; otherwise, it was considered a fluid node. Based on this procedure, two coarser representations of the 3D sample (NX = 200 and NX = 100) were generated, and the corresponding porosity and permeability values were evaluated for three different flow directions (FX+, FY+, and FZ+). The results are summarized in Table 7.

**Table 7:** Variation of porosity and directional permeability at different coarser resolutions in 3D simulations.

NX	Voxel size (μm)	Porosity (%)	Permeability k (D)		
			FX+	FY+	FZ+
400	18.7	69.8	561.9	249.1	798.3
200	37.4	67.7	593.7	245.8	885.3
100	74.8	70.9	1646.9	730.5	2573.5

It is observed that porosity remains nearly unchanged across different resolutions, whereas permeability increases as the resolution becomes coarser. In porous media, permeability is strongly controlled by critical constrictions; therefore, coarsening may introduce non-negligible errors in transport properties, even if global porosity remains similar. The significant variation in permeability values can be attributed to the artificial enlargement of throats during coarsening, which enhances flow capacity. These findings suggest that coarse simulations at NX = 100 are not suitable for accelerated simulations of this sample, as they fail to preserve the critical pore–throat structures controlling fluid transport. In contrast, convergence is considered achieved at NX = 400, as the results are of the same order of magnitude as those at NX = 200, with only minor differences.

## 5 Conclusion

This study has combined lattice Boltzmann simulations with micro-CT experiments to investigate fluid flow within a real bread structure, analyzing the impact of structural parameters on internal fluid dynamics.

From this analysis, it is observed that relying solely on (effective) porosity is insufficient to accurately predict permeability in the cereal-based foam structure. Pore connectivity — particularly in the direction of the applied force — was found to be the key factor controlling permeability. Well-connected pore networks reduce flow resistance and dead zones, thereby enhancing transport efficiency. In addition, pore size distribution also plays a crucial role in governing flow behavior. While larger pores facilitate higher fluid throughput, the efficiency of fluid transport is significantly influenced by the size and spatial arrangement of small, broken pores that act as bottlenecks, increasing flow resistance and pressure drop, and consequently reducing permeability. The median pore size ($D_{50}$) was found to be a representative scale for evaluating flow capacity. In contrast, tortuosity and the number of triple or quadruple junctions show only negligible correlations with permeability. These findings explain why even extended equations fail to accurately predict permeability, as they often neglect critical topological characteristics. Permeability predictions obtained from Lattice Boltzmann (LB) simulations incorporating real CT images could be a valuable tool for the effective characterization of porous structures. It is worth noting that the lattice Boltzmann Method offers clear advantages for flow simulations in porous media; however, it also involves certain trade-offs when steady-state solutions are required, as the method must evolve through a transient phase. Regarding advantages, LBM is inherently parallelizable and a very efficient implementation on GPU is possible. Additionally, LBM eliminates the need for complex meshing procedures, and the bounce-back boundary condition further simplifies the treatment of solid boundaries, allowing complex geometries to be accurately represented on an orthogonal lattice. These advantages have made LBM widely used for simulating both single-phase and multiphase flows in complex pore spaces. Since we are ultimately interested in bread penetration by sauces — an intrinsically time-dependent process — the necessity of transient simulations is not a real disadvantage.

This study represents the first step toward investigating sauce penetration in bread using the LB method. In future work, the LB model will be extended to capture realistic sauce–bread interactions, and additional influencing factors will be explored. On the one hand, non-Newtonian LB models will be developed and applied to investigate the effects of the power-law index and yield stress of sauces on fluid penetration in bread. On the other hand, two-phase LB models will be developed and applied to examine the influence of multiphase interactions on this penetration process. Furthermore, a systematic evaluation of different image processing methods will be considered to enhance microstructural accuracy in our future work. Meanwhile, comprehensive investigations of flow dynamics across multiple 3D samples will become the focus of our future work.

## Declaration of competing interest

The authors declare that they have no known competing financial interests or personal relationships that could have appeared to influence the work reported in this paper.
